# Emotional experience in the mornings and the evenings: consideration of age differences in specific emotions by time of day

**DOI:** 10.3389/fpsyg.2014.00185

**Published:** 2014-03-06

**Authors:** Tammy English, Laura L. Carstensen

**Affiliations:** ^1^Department of Psychology, Washington University in St. LouisSt. Louis, MO, USA; ^2^Department of Psychology, Stanford UniversityStanford, CA, USA

**Keywords:** affect, emotional experience, discrete emotions, aging, circumplex model

## Abstract

Considerable evidence points to age-related improvements in emotional well-being with age. In order to gain a more nuanced understanding of the nature of these apparent shifts in experience, we examined age differences in a range of emotional states in the mornings and evenings in a sample of 135 community-residing participants across 10 consecutive days. Participants ranged in age from 22 to 93 years. Each participant completed a diary in the morning and again in the evening every day for the study period. During each of the assessments, participants reported the degree to which they experienced emotions sampled from all four quadrants of the affective circumplex. Overall, participants felt less positive and more negative in the evenings than in the mornings. As expected, older adults reported a relatively more positive emotional experience than younger adults at both times of day. Importantly, however, age effects varied based on emotion type and time of day. Older adults reported experiencing more positive emotion than relatively younger adults across a range of different positive states (although age differences emerged most consistently for low arousal positive states). Age-related reductions in negative experience were observed only for reports of low arousal negative emotions. There were no age differences in anger, anxiety, or sadness. For some emotions, age differences were stronger in the mornings (e.g., relaxed) whereas for other emotions age differences were more pronounced in the evenings (e.g., enthusiastic). Findings are discussed in the context of adulthood changes in motivation and emotional experience.

## Inroduction

Emotional well-being appears to improve with age (see reviews by Consedine and Magai, 2006; Scheibe and Carstensen, 2010; Charles and Carstensen, 2013) and, importantly, age differences do not appear to be due to cohort effects since longitudinal evidence also points to improvements in emotional experience within individuals as they grow older (Charles et al., 2001; Carstensen et al., 2011). In order to gain a more nuanced understanding of age-differences in everyday life, in the present study we examined whether age differences in emotional experience are apparent across a range of emotions and at different points in the day.

One of the first reports of age-related improvements in emotional functioning was published by Mroczek and Kolarz (1998). Based on a cross-sectional comparison in a large representative sample of Americans, they observed that age was associated with more reported positive experience and less negative experience. Many replications of overall improvements in emotional experience have followed, although findings have been less consistent concerning the emotional changes that drive improvements in emotional well-being. Some studies find that age differences are driven by reductions in negative emotional experience (e.g., Carstensen et al., 2000) whereas others observe the differences to be due to increases in positive emotional experience (e.g., Riediger et al., 2009). In a very large scale cross-sectional study based on yesterday interviews^1^, Stone et al. (2010) observed a peak in happiness in early old age, followed by relative constancy into more advanced ages, while negative emotions showed linear declines. A handful of large-scale studies have found small age-related increases in depressive symptoms (Fiske et al., 2003; Davey et al., 2004) and negative emotional experiences (Diener and Suh, 1998); however, studies that have controlled for physical health typically eliminate these effects suggesting that illness more than age is responsible for such findings (Gatz et al., 1996; Kunzmann et al., 2000; although see Fiske et al., 2003).

One explanation for differences in the published literature reflects the fact that researchers use a wide range of measures and procedures, which tap different aspects of emotional experience. While the variety in approaches adds support for the contention that emotional well-being improves with age, the same variability may account for discrepancies across studies in patterns of findings. Global measures, for example, tap deliberative evaluations of life more than emotional experience *per se*. Experience sampling, in contrast, typically considered the gold standard in the measurement of emotion in daily life, does an excellent job of characterizing daily life yet often misses low base rate emotions like surprise and anger. Thus, though experience sampling characterizes the emotional tone of daily life very well (most people are not routinely surprised, for example), it can downplay the role of short-lived but powerful emotional experiences. These types of low frequency emotional states might be better detected by global assessments or daily diary studies that ask individuals to reflect on specific experiences across an entire day. Memory based measures, such as, yesterday interviews, are susceptible to motivated reconstructions and can be influenced differentially by motivated processes (e.g., positivity effect, Mather and Carstensen, 2005; Reed and Carstensen, 2012).

The measures researchers adopt also vary in the degree to which they tap high and low arousal. Affective states have been found to vary along two independent dimensions: valence and arousal (Watson and Tellegen, 1985; Larsen and Diener, 1992). Excitement, for example, is both positive and arousing whereas contentment is positive and low in arousal. Traditionally, widely used measures of experience developed for use with young populations have focused mostly on high arousal emotional states, especially in regard to positive emotion (e.g., excitement, enthusiasm), creating an inadvertent conflation of arousal and valence.

These measurement issues are particularly troublesome because increasingly findings point to arousal as a key aspect of emotional development. Powell Lawton et al. (1992) observed many years ago that older adults are less likely to report high arousal or surgent emotions. Consistent with this observation, Kessler and Staudinger (2009) recently reported findings from a study comparing younger and older adults where age differences were observed when emotional composites were broken down by valence and arousal. Specifically, older adults only reported more low arousal positive states (e.g., relaxed), whereas they reported less negative emotion regardless of valence. In an experience sampling study, Scheibe et al. (2013) also observed age-related increases in the daily experience of low arousal positive emotion but no age differences for high arousal positive states.

One potential explanation for these findings comes from the strengths and vulnerabilities integration model (SAVI; Charles, 2010), which suggests that older adults avoid situations that elicit high arousal states because they are less able to recover from disruptions of physiological homeostasis. Therefore, as individuals age they may come to focus their emotional goals more on activities that engender low positive arousal states (e.g., calm) and avoid high arousal states, regardless of valence. Indeed, Scheibe et al. (2013), noted above, found that older adults reported a greater preference for low arousal positive emotions relative to high arousal positive emotions than younger adults. In addition, age-related decreases in negative emotions have been found consistently for high arousal negative states (e.g., anger, rage, despair; Lawton et al., 1992; Schieman, 1999; Phillips et al., 2008; Ross and Mirowsky, 2008; Stone et al., 2010), but it is less clear whether there are similar declines in the experience of low arousal negative states such as sadness. Thus, in order to better understand how emotional experience changes with age, it will be necessary to decompose emotion composites into more fine grained emotional states. Based on the extant literature, emotional benefits associated with age seem to come primarily in the form of increased experience of low arousal positive affective states coupled with a decrease in negative emotions, particularly those that are arousing (e.g., anger; Stone et al., 2010).

Another potential source of inconsistency in the literature on age differences in emotion is time of day. Studies have typically focused on either global reports of emotional experience (typically or on a given day) or momentary emotional experience averaged across various time points (in a day or a series of days). It is conceivable that age differences are stronger in the mornings than evening. Prior research suggests that cognitive performance varies by time of day, such that older adults perform better early in the day whereas younger adults perform better later in the day (Yoon et al., 1999; Knight and Mather, 2013). Emotion regulation, like many goal-directed processes, often demands cognitive resources (Muraven et al., 1998; Ochsner et al., 2012), so if emotion regulation plays a role in older adults relatively more positive emotional experience, then time of day may influence age differences in emotional experience as well. That is, it is conceivable that widely documented emotional advantages of older adults are strongest in the mornings. To our knowledge, however, although older people are more likely to describe themselves as “morning types” than younger adults (Biss and Hasher, 2012), age differences in emotion per se have not been examined as a function of time of day.

In order to gain a clearer picture of age differences in emotional life, we examined a range of specific emotions representing all four quadrants in the affective circumplex, in the mornings and in the evenings across a 10-day period. We hypothesized that older adults would show a more positive affective profile overall and, based on prior research, that older adults would report relatively more low arousal positive emotion and less high arousal negative emotion. We also hypothesized that age differences in emotional experience would be more pronounced in the mornings, consistent with prior research on cognitive performance. In addition, we examined two potential sources of differential age differences in morning versus evening emotional experience: sleep and individual differences in morningness. Older adults may report relatively more positive emotional experience in the morning because they are more likely to be “morning types” or have different sleep patterns than younger adults.

## Methods

### Participants

The sample consisted of 135 participants (50% female) ranging in age from 22 to 93 years (*M* = 53.87, *SD* = 19.23). Participants were roughly evenly distributed across this age range. Sixty-seven percent of the participants were European American, 30% were African American, and 3% identified with multiple ethnicities. Socioeconomic status (SES) was determined by a survey research firm based on answers to questions about income, education and occupation. Thirty-two percent of participants were blue-collar workers, 64% were white-collar workers, and 4% were pink-collar workers. Gender, ethnicity, and SES were stratified across age.

### Procedure

After providing informed consent, participants completed a number of measures, including assessments of health, cognition, and morningness. The experimenter then explained the format and schedule of the daily diaries. For 10 consecutive days, participants completed an online diary in the morning and again in the evening. Participants were asked to complete the diaries as soon as they woke up and just before bedtime in the evening. Emotional experience was assessed at both time points (morning and evening) each day; sleep the previous night was assessed only in the morning. Reminder emails were sent each day around the time participants would be completing the diaries. Participants accessed the online diaries by logging into a secure website where they were presented with a series of questions. Prior to recruitment, this study was approved by the Institutional Review Board (IRB) at Stanford University.

### Materials

#### Daily emotional experience

In each of the morning and evening diaries, participants rated on a 7-point scale that ranged from *not at all* to *extremely* the degree to which they were currently feeling each of 14 emotions. The list of emotions included terms commonly used in emotion research and covered each quadrant of the affective circumplex. Specifically, there were 7 positive emotions, including happiness (a prototypical pleasant emotion), 3 high arousal states (enthusiastic, excited, proud), and 3 low arousal states (calm, content, relaxed), as well as 7 negative emotions, including sadness (a prototypical unpleasant emotion), 2 high arousal states (angry and anxious/worried), 2 low arousal states (bored and fatigued), and 2 other states of particular interest in old age (lonely and regretful). These emotion terms were presented together on one screen in a randomized order at each assessment point.

#### Daily sleep

At each morning assessment, participants rated the quality of their sleep the previous night (1 = *very bad* to 7 = *very good*) and indicated their subjective sleep quantity by rating how much they slept the previous night (1 = *not at all* to 5 = *a lot*). They also listed the number of hours they slept the previous night.

#### Physical health

Participants completed a comprehensive health questionnaire (Hultsch et al., 1993) that assessed multiple facets of physical health, including self-rated overall health (*M* = 3.15, *SD* = 1.22), illness episodes (*M* = 2.41, *SD* = 1.82), instrumental health (*M* = 1.28, *SD* = 2.63), chronic illnesses (weighted by severity rating, *M* = 1.92, *SD* = 2.15), and number of medications (*M* = 1.51, *SD* = 1.54). An index of physical health was computed by averaging scores across these five dimensions (alpha = 0.70). Higher scores indicate poorer physical health.

#### Verbal fluency

To assess verbal fluency, participants were asked to verbally list as many animals as possible in 90 s. This is a widely used measure that has been shown to be strongly associated with general intellectual ability (Lindenberger et al., 1993).

#### Morningness

Individual differences in morningness were assessed using the 5-item brief Morningness-Eveningness Questionnaire (MEQ; Adan and Almirall, 1991). This scale is well-validated and has been extensively used in younger and older adults samples. An example item is, “At what time of the day do you think that you reach your “feeling best” peak?” To determine overall ratings of morningness-eveningness, each question is given a scaled score and then these scores are summed. Higher scores indicate greater morningness.

## Results

### Data reduction and analytic approach

#### Emotion indices

The data we report represent the intensity of emotion reports^2^. In addition to examining specific emotions, we computed composites to represent the degree to which participants reported positive and negative emotional experience on each occasion. The positive emotion composite was created by averaging momentary ratings across calm, content, enthusiastic, excited, happy, proud, and relaxed (alpha= 0.89). The negative emotion composite was created by averaging momentary ratings across angry, anxious/worried, bored, fatigued, sad, regretful, and lonely (alpha= 0.83). Descriptive statistics for these emotion composites and the specific emotions appear in Table 1.

**Table 1:** **Descriptive statistics for emotional experience in the morning and the evening**.

	**Intensity of experience**				**Frequency of experience**			
	**Mean**	***SD***	**Min.**	**Max.**	**Mean**	***SD***	**Min.**	**Max.**
**AM POSITIVE**	**4.36**	**0.94**	**1.74**	**6.77**	**0.94**	**0.10**	**0.46**	**1.00**
Happy	4.74	1.13	1.33	7.00	0.97	0.11	0.22	1.00
Content	4.88	1.08	1.50	7.00	0.98	0.08	0.30	1.00
Calm	4.88	1.01	2.10	7.00	0.98	0.06	0.60	1.00
Relaxed	4.71	1.09	2.07	7.00	0.98	0.05	0.71	1.00
Enthusiastic	3.98	1.29	1.00	7.00	0.92	0.19	0.00	1.00
Excited	3.56	1.26	1.00	6.80	0.87	0.23	0.00	1.00
Proud	3.79	1.52	1.00	7.00	0.87	0.25	0.00	1.00
**AM NEGATIVE**	**1.76**	**0.63**	**1.00**	**3.99**	**0.35**	**0.27**	**0.00**	**1.00**
Sad	1.48	0.66	1.00	4.10	0.26	0.32	0.00	1.00
Bored	1.46	0.67	1.00	3.70	0.25	0.32	0.00	1.00
Fatigued	2.47	1.16	1.00	6.10	0.61	0.37	0.00	1.00
Anxious	2.37	1.16	1.00	5.60	0.57	0.37	0.00	1.00
Angry	1.32	0.54	1.00	4.00	0.17	0.27	0.00	1.00
Lonely	1.59	0.78	1.00	4.90	0.31	0.34	0.00	1.00
Regretful	1.62	0.98	1.00	5.30	0.29	0.37	0.00	1.00
**PM POSITIVE**	**4.30**	**0.92**	**2.06**	**6.57**	**0.93**	**0.11**	**0.49**	**1.00**
Happy	4.84	1.10	1.60	6.90	0.98	0.07	0.44	1.00
Content	4.90	1.06	1.30	7.00	0.98	0.08	0.30	1.00
Calm	4.86	1.04	2.22	7.00	0.98	0.07	0.60	1.00
Relaxed	4.74	1.04	1.78	7.00	0.98	0.06	0.56	1.00
Enthusiastic	3.57	1.31	1.00	6.60	0.88	0.22	0.00	1.00
Excited	3.24	1.22	1.00	6.40	0.83	0.26	0.00	1.00
Proud	3.93	1.47	1.00	7.00	0.89	0.23	0.00	1.00
**PM NEGATIVE**	**1.89**	**0.68**	**1.00**	**4.04**	**0.38**	**0.26**	**0.00**	**1.00**
Sad	1.56	0.81	1.00	5.36	0.27	0.33	0.00	1.00
Bored	1.58	0.81	1.00	4.20	0.30	0.35	0.00	1.00
Fatigued	3.23	1.39	1.00	6.60	0.76	0.32	0.00	1.00
Anxious	2.17	1.08	1.00	6.00	0.53	0.37	0.00	1.00
Angry	1.41	0.61	1.00	3.71	0.20	0.28	0.00	1.00
Lonely	1.69	1.05	1.00	5.40	0.30	0.38	0.00	1.00
Regretful	1.60	0.82	1.00	4.70	0.30	0.35	0.00	1.00

AM indicates the morning assessment and PM indicates the evening assessment. Intensity of experience represents average emotion ratings across the 10-day sampling period. Frequency of emotion represents the percent of occasions that participants reported experiencing an emotion to any degree (i.e., greater than the 1 on the rating scale) across the 10-day sampling period.

#### Analytic approach

To examine age differences in daily emotion, we conducted multilevel modeling using the linear MIXED MODELS function in SPSS. We tested a three-level model, in which time of day (AM or PM) was nested within days and days were nested within persons. Maximum likelihood (ML) estimation was used to account for missing data. Age effects were tested with linear and quadratic age terms. Time of day (morning = 0, evening = 1) and its interaction with each of the age components were also included as predictors. Age was correlated with poorer physical health (*r* = 0.25, *p* < 0.05) and lower verbal fluency (*r* = −0.20, *p* < 0.05), so health and fluency were included in the models as time invariant covariates. Results are reported as unstandardized MLM coefficients in Table 2.

**Table 2:** **Results of multilevel analyses testing the effects of age and time of day on daily emotion**.

**Fixed effects estimates**	**Intercept**	**Age**	**Age^2^**	**Time of Day**	**Time × Age**	**Time × Age^2^**	**Health**	**Fluency**
**POSITIVE**								
Happy	4.7056 (0.1309)	0.0140 (0.0048)^**^	0.0000 (0.0003)	−0.0063 (0.0496)	−0.0005 (0.0018)	0.0003 (0.0000)^**^	−0.5137 (0.1347)^**^	−0.1205 (0.0886)
Content	4.8204 (0.1281)	0.0153 (0.0047)^**^	0.0002 (0.0003)	−0.0716 (0.0525)	−0.0027 (0.0019)	0.0002 (0.0001)^*^	−0.4009 (0.1315)^**^	0.0078 (0.0865)
Calm	4.9099 (0.1225)	0.0151 (0.0045)^**^	0.0000 (0.0002)	−0.0903 (0.0575)	−0.0023 (0.0020)	0.0002 (0.0001)^+^	−0.3326 (0.1248)^**^	0.0381 (0.0821)
Relaxed	4.8582 (0.1267)	0.0205 (0.0047)^**^	−0.0004 (0.0003)	−0.0944 (0.0591)	−0.0086 (0.0021)^**^	0.0004 (0.0001)^**^	−0.3029 (0.1291)^*^	0.0262 (0.0850)
Enthusiastic	4.0590 (0.1472)	0.0225 (0.0054)^**^	−0.0002 (0.0003)	−0.5139 (0.0576)^**^	0.0050 (0.0020)^*^	0.0003 (0.0001)^*^	−0.4827 (0.1513)^**^	−0.0713 (0.0995)
Excited	3.5770 (0.1492)	0.0048 (0.0055)	0.0000 (0.0003)	−0.3560 (0.0587)^**^	0.0040 (0.0021)^+^	0.0000 (0.0001)	−0.4265 (0.1534)^**^	−0.1834 (0.1009)^+^
Proud	3.9011 (0.1827)	0.0062 (0.0067)	−0.0003 (0.0004)	0.1625 (0.0410)^**^	−0.0044 (0.0018)^*^	0.0000 (0.0001)	−0.2324 (0.1897)	−0.3412 (0.1248)^*^
**NEGATIVE**								
Sad	1.5833 (0.0912)	−0.0022 (0.0034)	−0.0003 (0.0002)	0.1346 (0.0423)^**^	−0.0027 (0.0015)^+^	−0.0002 (0.0000)^+^	0.0761 (0.0930)	0.0432 (0.0612)
Bored	1.4836 (0.0919)	−0.0068 (0.0034)^*^	0.0000 (0.0002)	0.1050 (0.0390)^**^	−0.0021 (0.0014)	0.0000 (0.0000)	0.0268 (0.0941)	−0.0327 (0.0619)
Fatigued	2.4868 (0.1453)	−0.0145 (0.0053)^**^	0.0000 (0.0003)	0.6548 (0.0705)^**^	−0.0006 (0.0025)	0.0003 (0.0001)^*^	0.5508 (0.1478)^**^	0.1630 (0.0972) ^+^
Anxious	2.3964 (0.1396)	−0.0065 (0.0051)	0.0000 (0.0003)	−0.1869 (0.0573)^**^	0.0017 (0.0020)	0.0000 (0.0001)	0.0250 (0.1432)	−0.0698 (0.0942)
Angry	1.3914 (0.0703)	−0.0047 (0.0026)^+^	−0.0002 (0.0001)	0.1341 (0.0420)^**^	−0.0015 (0.0015)	−0.0001 (0.0000)	0.1044 (0.0703)	−0.0590 (0.0463)
Lonely	1.6722 (0.1276)	−0.0029 (0.0047)	−0.0001 (0.0003)	0.0540 (0.0427)	−0.0011 (0.0015)	0.0000 (0.0000)	0.0802 (0.1319)	−0.0626 (0.0868)
Regretful	1.7191 (0.1002)	−0.0052 (0.0037)	−0.0004 (0.0002)^+^	0.0021 (0.0431)	−0.0005 (0.0015)	0.0000 (0.0000)	0.1490 (0.1026)	−0.0062 (0.0675)

Unstandardized estimates are presented with standard errors in parentheses. Age is centered at age 54 years; Time of day coded 0 for morning, and 1 for evening; Physical health and verbal fluency Z-scored.

+ p < 0.10;

* p < 0.05;

** p < 0.01.

To better understand potential interactions between time of day and age, we also fit a model with random intercept, linear age, and quadratic age components separately for morning and evening emotion (controlling for physical health and verbal fluency). Results are reported as unstandardized MLM coefficients in Table 3.

**Table 3:** **Results of multilevel analyses testing the effects of age on morning and evening emotion**.

**Fixed effects estimates**	**Intercept**	**Age**	**Age^2^**	**Health**	**Fluency**
**AM POSITIVE**					
Happy	4.7033 (0.1332)	0.0136 (0.0049)^**^	0.0001 (0.0003)	−0.5104 (0.1396)^**^	−0.1597 (0.0919)^+^
Content	4.8159 (0.1308)	0.0144 (0.0048)^**^	0.0002 (0.0003)	−0.3684 (0.1371)^**^	−0.0542 (0.0902)
Calm	4.9092 (0.1219)	0.0152 (0.0045)^**^	0.0000 (0.0002)	−0.3459 (0.1278)^**^	0.0234 (0.0841)
Relaxed	4.8576 (0.1282)	0.0201 (0.0047)^**^	−0.0004 (0.0003)	−0.3032 (0.1344)^*^	−0.0024 (0.0885)
Enthusiastic	4.0589 (0.1510)	0.0224 (0.0056)^**^	−0.0002 (0.0003)	−0.4996 (0.1583)^**^	−0.0846 (0.1042)
Excited	3.5805 (0.1547)	0.0046 (0.0057)	0.0000 (0.0003)	−0.4338 (0.1621)^**^	−0.1849 (0.1067)^+^
Proud	3.9012 (0.1853)	0.0058 (0.0068)	−0.0003 (0.0004)	−0.2404 (0.1943)	−0.3898 (0.1278)^**^
**AM NEGATIVE**					
Sad	1.5829 (0.0825)	−0.0024 (0.0030)	−0.0003 (0.0002)^+^	0.0666 (0.0864)	0.0223 (0.0569)
Bored	1.4816 (0.0832)	−0.0063 (0.0031)^*^	0.0000 (0.0002)	−0.0048 (0.0872)	−0.0241 (0.0574)
Fatigued	2.4868 (0.1410)	−0.0137 (0.0052)^*^	0.0000 (0.0003)	0.4161 (0.1478)^**^	0.0954 (0.0973)
Anxious	2.3930 (0.1463)	−0.0064 (0.0054)	0.0000 (0.0003)	−0.0142 (0.1534)	−0.1155 (0.1009)
Angry	1.3929 (0.0676)	−0.0045 (0.0025)^+^	−0.0002 (0.0001)	0.0713 (0.0709)	−0.0565 (0.0467)
Lonely	1.6725 (0.1247)	−0.0025 (0.0046)	−0.0001 (0.0003)	0.0526 (0.1307)	−0.0518 (0.0860)
Regretful	1.7168 (0.0974)	−0.0051 (0.0036)	−0.0004 (0.0002)^+^	0.1317 (0.1021)	−0.0179 (0.0672)
**PM POSITIVE**					
Happy	4.7009 (0.1299)	0.0139 (0.0048)^**^	0.0004 (0.0003)	−0.5158 (0.1361)^**^	−0.0812 (0.0896)
Content	4.7525 (0.1269)	0.0135 (0.0047)^**^	0.0004 (0.0003)	−0.4309 (0.1330)^**^	0.0689 (0.0875)
Calm	4.8197 (0.1270)	0.0128 (0.0047)^**^	0.0001 (0.0003)	−0.3149 (0.1331)^*^	0.0529 (0.0876)
Relaxed	4.7633 (0.1275)	0.0123 (0.0047)^*^	0.0000 (0.0003)	−0.2975 (0.1338)^*^	0.0561 (0.0880)
Enthusiastic	3.5436 (0.1511)	0.0275 (0.0056)^**^	0.0000 (0.0003)	−0.4644 (0.1584)^**^	−0.0572 (0.1042)
Excited	3.2169 (0.1486)	0.0089 (0.0055)	0.0000 (0.0003)	−0.4192 (0.1557)^**^	−0.1818 (0.1025)^+^
Proud	4.0631 (0.1817)	0.0023 (0.0067)	−0.0004 (0.0004)	−0.2238 (0.1905)	−0.2921 (0.1254)^*^
**PM NEGATIVE**					
Sad	1.7190 (0.1004)	−0.0047 (0.0037)	−0.0004 (0.0002)^*^	0.0848 (0.1052)	0.0645 (0.0693)
Bored	1.5902 (0.1007)	−0.0093 (0.0037)^*^	0.0000 (0.0002)	0.0584 (0.1055)	−0.0410 (0.0695)
Fatigued	3.1408 (0.1633)	−0.0158 (0.0060)^*^	0.0002 (0.0003)	0.6816 (0.1711)^**^	0.2306 (0.1126)^*^
Anxious	2.2126 (0.1371)	−0.0048 (0.0051)	−0.0001 (0.0003)	0.0631 (0.1437)	−0.0228 (0.0946)
Angry	1.5247 (0.0752)	−0.0065 (0.0028)^*^	−0.0003 (0.0002)^+^	0.1365 (0.0787)^+^	−0.0611 (0.0519)
Lonely	1.7270 (0.1325)	−0.0044 (0.0049)	−0.0001 (0.0003)	0.1071 (0.1390)	−0.0734 (0.0914)
Regretful	1.7227 (0.1028)	−0.0057 (0.0038)	−0.0003 (0.0002)	0.1638 (0.1077)	0.0054 (0.0709)

Unstandardized estimates are presented with standard errors in parentheses. Age is centered at age 54 years; Physical health and verbal fluency Z-scored.

+ p < 0.10;

* p < 0.05;

** p < 0.01.

In preliminary analyses, we tested for effects of gender, SES and ethnicity on positive and negative emotion. There were no main effects of these demographic variables in predicting emotional experience, nor did they moderate the effects of age on emotion. However, there were gender differences in the effects of Time of Day for both positive emotion (Gender × Time of Day = −0.1674, *p* < 0.05) and negative emotion (Gender × Time of Day = 0.1899, *p* < 0.01). Compared to the morning, women reported feeling less positive emotion (γ = −0.2196, *p* < 0.01) and more negative emotion in the evening (γ = 0.2220, *p* < 0.01); however, there were no Time of Day effects for men (positive emotion: γ = −0.0521, *p* = 0.24; negative emotion: γ = 0.0321, *p* = 0.35). In addition, for negative emotion, the interaction between age and time of day varied based on gender (Gender × Age^2^ × Time of Day: γ = −0.0004, *p* < 0.05; Age^2^ × Time of Day for men: γ = 0.0002, *p* < 0.01; Age^2^ × Time of Day for women: γ = −0.0002, *p* < 0.05), as well as based on SES (SES × Age × Time of Day: γ = −0.0056, *p* < 0.01) and ethnicity (Ethnicity × Age × Time of Day: γ = 0.0058, *p* < 0.01). Age moderated the effect of Time of Day on negative emotion among African-Americans (γ = −0.0045, *p* < 0.05) and blue collar workers (γ = −0.0047, *p* < 0.01), but not among European-Americans (γ = 0.0002, *p* = 0.86) and white collar workers (γ = 0.0009, *p* = 0.39); specifically, the tendency for participants to feel more negative emotion in the evening than in the morning was attenuated among older African-Americans and older blue-collar workers.

### Age effects

Age was linearly associated with more overall positive emotion (γ = 0.0141, *p* < 0.01) and less negative emotion (γ = −0.0061, *p* < 0.05). In terms of positive specific emotions, older adults reported feeling more happy (γ = 0.0140, *p* < 0.01) and more of each of the low arousal positive emotions, including content (γ = 0.0153, *p* < 0.01), calm (γ = 0.0151, *p* < 0.01), and relaxed (γ = 0.0205, *p* < 0.01). In addition, there was an age-related increase in enthusiasm (γ = 0.0225, *p* < 0.01), but there were not significant age differences for the two other higher arousal positive emotions (excitement: γ = 0.0048, *p* = 0.39; pride: γ = 0.0062, *p* = 0.36). In terms of specific negative emotions, older adults only reported feeling less bored (γ = −0.0068, *p* < 0.05) and less fatigued (γ = −0.0145, *p* < 0.01) than did younger adults. Age was unrelated to reports of sadness (γ = −0.0022, *p* = 0.51), loneliness (γ = −0.0029, *p* = 0.54), and regret (γ = −0.0052, *p* = 0.16), as well as high arousal negative emotions, anxiety (γ = −0.0065, *p* = 0.21) and anger (γ = −0.0047, *p* = 0.069). There were no significant quadratic effects of age.

### Time of day effects

Participants reported less positive emotion (γ = −0.1372, *p* < 0.01) and more negative emotion (γ = 0.1288, *p* < 0.01) in the evenings compared to mornings. Specifically, compared to the mornings, in the evenings participants' reported less enthusiasm (γ = −0.5139, *p* < 0.01) and excitement (γ = −0.3560, *p* < 0.01), as well as more sadness (γ = 0.1346, *p* < 0.01), boredom (γ = 0.1050, *p* < 0.01), fatigue (γ = 0.6549, *p* < 0.01), and anger (γ = 0.1341, *p* < 0.01). Two exceptions to the general pattern of reduced well-being at the end of the day were pride and anxiety. Pride was experienced more (γ = 0.1625, *p* < 0.01) and anxiety was experience less (γ = −0.1869, *p* < 0.01) in the evenings than in the mornings. Happiness (γ = −0.0063, *p* = 0.90) and low arousal positive emotions (content: γ = −0.0716, *p* = 0.17; calm: γ = −0.0903, *p* = 0.12; relaxed: γ = −0.0944, *p* = 0.11) did not differ by time of day, nor did loneliness (γ = 0.0540, *p* = 0.21) or regret (γ = 0.0021, *p* = 0.96).

### Moderation of age effects by time of day

**Figure 1:**
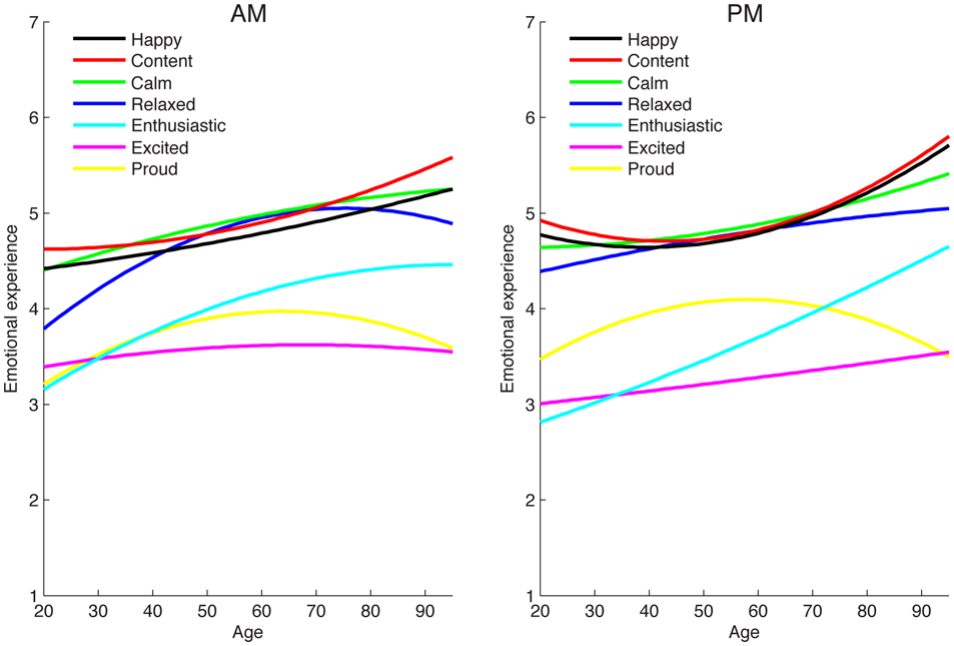
**Age differences in positive emotional experience in the morning (AM) and evening (PM)**.

**Figure 2:**
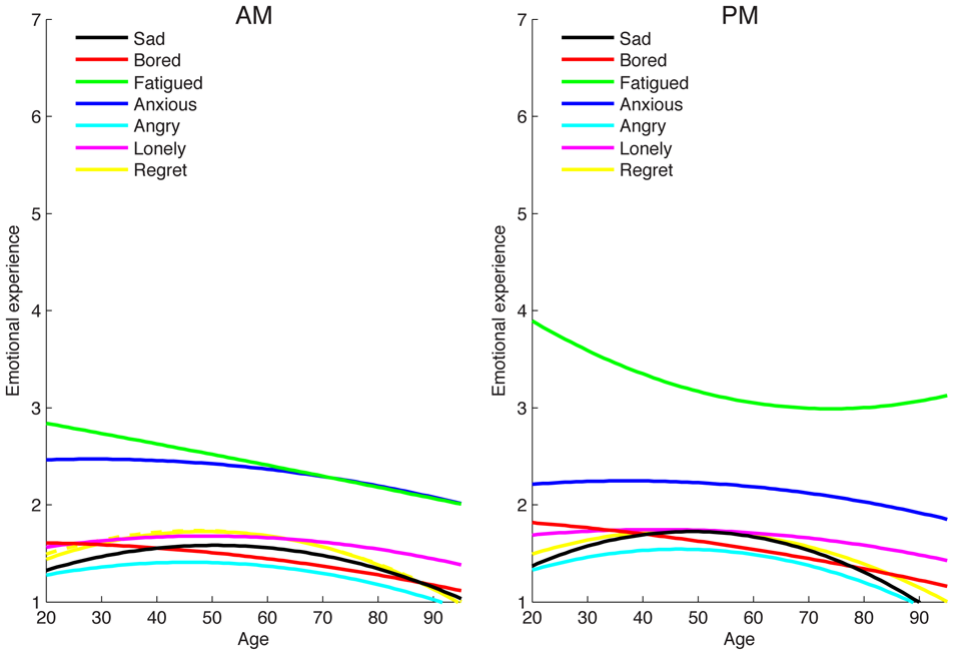
**Age differences in negative emotional experience in the morning (AM) and evening (PM)**.

The effect of age on emotional experience varied somewhat as a function of when emotion as assessed, especially for positive states. There were significant interactions between time of day and age for all of the positive emotions (although the effects were only marginal for calm and excited). However, the nature of this interaction varied across the specific emotions. For instance, as shown in Figure 1, older adults enhanced experience of relaxation was greater in the morning than the evening (Time of Day × Age: γ = −0.0086, *p* < 0.01), whereas their increased experience of enthusiasm showed the reverse pattern (i.e., it was stronger in the evening; Time of Day × Age: γ = 0.0050, *p* < 0.05). Notably, as shown in Table 3, at both times of day older adults reported feeling significantly more relaxed (morning: γ = 0.0201, *p* < 0.01; evening: γ = 0.0123, *p* < 0.05) and enthusiastic (morning: γ = 0.0224, *p* < 0.01; evening: γ = 0.0275, *p* < 0.05) than younger people. Time also moderated the effect of age on happiness (Time of Day × Age^2^: γ = 0.0003, *p* < 0.01) and contentment (Time of Day × Age^2^: γ = 0.0002, *p* < 0.05), such that there was more of a quadratic (accelerated) age effect in the evening than in the morning (see Figure 2). However, there were significant linear effects of age on happiness and contentment at both time points (no quadratic age effects): older adult felt happier and more content than relatively younger adults in both the mornings (happy: γ = 0.0136, *p* < 0.01; content: γ = 0.0144, *p* < 0.01) and in the evenings (happy: γ = 0.0139, *p* < 0.01; content: γ = 0.0135, *p* < 0.01). For pride, the effect of age was weaker in the evening than in the morning (γ = −0.0044, *p* < 0.05), but there were not significant age differences at either point in the day (morning: γ = 0.0058, *p* = 0.40; evening: γ = 0.0023, *p* = 0.74). Both younger and older people experienced more pride in the evenings than mornings.

Age differences in negative emotion varied less based on time of assessment. There was only a significant interaction for fatigue (Time × Age^2^: γ = 0.0003, *p* < 0.05). As shown in Figure 2, the age-related decrease in fatigue flattened at advanced ages for evening emotion more so than for morning emotion (although there were significant linear age effects at both time points). For sadness, age effects were somewhat stronger in the evening: there was a significant quadratic effect of age in the evening (Age^2^: γ = −0.0004, *p* < 0.05) but this effect was not significant in the morning (Age^2^: γ = −0.0003, *p* = 0.09). However, there was not a significant interaction between time of day and age for sadness (Time × Age: γ = −0.0027, *p* = 0.07; Time × Age^2^: γ = −0.0002, *p* = 0.07). Regardless of age and time of day, individuals reported relatively low levels of sadness (see Figure 2). There were not significant interactions between time of day and age for the other negative emotions either, including the higher arousal negative states (anger and anxiety).

### Ancillary analyses: sleep and morningness

We ran additional models that included the measures of sleep (quantity and quality the previous night) and individual differences in morning along with the core predictors (i.e., linear and quadratic age terms, Time of Day, and the interaction between Time of Day and each of the age components) to test whether these variables might explain the effects of Age or the interaction between Age and Time of Day. The sleep variables were included together as day-level predictors; morningness was included as a person-level predictor along with its interaction with Time of Day.

Older adults reported better subjective sleep quantity (Age: γ = 0.0091, *p* < 0.001) and quality (Age: γ = 0.0109, *p* < 0.01); for number of hours slept, there was a quadratic age effect indicating that middle-aged adults slept fewer hours than did relatively younger and older adults (Age: γ = −0.0049, *p* = 0.27; Age^2^: γ = 0.0008, *p* < 0.01). Individuals who reported better sleep quality the previous night felt more of each of the positive emotions (happy: γ = 0.0795, *p* < 0.01; content: γ = 0.0849, *p* < 0.01; calm: γ = 0.1062, *p* < 0.001; relaxed: γ = 0.0867, *p* < 0.01; enthusiastic: γ = 0.0882, *p* < 0.01; excited: γ = 0.0920, *p* < 0.01; pride: γ = 0.0592, *p* < 0.05) and less of each of the negative emotion (sadness: γ = −0.1059, *p* < 0.001; anger: γ = −0.0442, *p* < 0.05; boredom: γ = −0.0398, *p* < 0.05; fatigue: γ = −0.1713, *p* < 0.001; lonely: γ = −0.0572, *p* < 0.01; regret: γ = −0.0732, *p* < 0.01) except anxiety (γ = −0.0474, *p* = 0.083). Importantly, however, the effects of Age and the Age × Time of Day interactions remained intact when the sleep variables were included as covariates. Only the age effect for boredom became marginal when controlling for sleep (γ = −0.0065, *p* = 0.059).

As expected, older adults scored higher on morningness (*r* = 0.40). However, when both Age and Morningness were included as predictors of emotional experience, all the effects of Age and the Age × Time of Day interactions remained significant. The only significant main effect of Morningness was for anger: individuals higher on morningness reported feeling less angry (γ = −0.1052, *p* < 0.05). There were also significant Morningness × Time of Day interactions for calm (γ = 0.1135, *p* < 0.01), relaxed (γ = 0.1013, *p* < 0.05), and enthusiastic (γ = −0.0861, *p* < 0.05): individuals higher in morningness reported feeling relatively more calm and relaxed in the evening (compared to the morning) and more enthusiastic in the morning (compared to the evening). Overall, these findings suggest that sleep patterns and individual differences in morningness do not explain the age differences in emotional experience observed in this study.

## Discussion

Consistent with previous findings, older adults in the present study reported relatively more positive daily emotional experiences than did younger adults. Older adults characterized their emotional experiences as more positive and less negative in both the mornings and evenings. This finding contributes to a compelling literature on age-related improvements in the emotion domain. Older adults' relatively greater emotional well-being has been observed across a wide range of samples and in studies using a wide variety of methods. Whether emotions are sampled randomly throughout the course of a week, studied in the laboratory or recalled at the end of the day or from the day before, age advantages reliably appear. Evidence about the specific motions that drive these age differences is far more mixed. In some studies, greater positive experience accounts for age differences. In others, less negative experience accounts for differences. Findings from several recent studies suggest that these variations may be due to differences in methodological approaches and the measures used to assess emotion.

In the present study, we aimed to capture a more differentiated view of emotional experience across specific positive and negative emotions early and late in the day. We expected that age differences would be more pronounced for low arousal emotions, given previous findings (Kessler and Staudinger, 2009; Scheibe et al., 2013) and theory (Charles, 2010), specifically contentions stemming from the strengths and vulnerabilities integration model (SAVI) that high arousal states may be unpleasant for older adults. Sampling a range of emotions that represented all four quadrants of the affective circumplex, we asked participants spanning in age from young adulthood to very old age to describe how calm, content, enthusiastic, excited, happy, proud, relaxed, angry, anxious/worried, bored, fatigued, sad, regretful, and lonely they felt every morning and evening across ten days. The hypothesis that age differences would be most apparent for low arousal positive emotions was largely supported. Of the seven positive emotions sampled, older people reported greater experience of all of them except two high arousal emotions, namely excitement and pride. However, among negative emotions, only two distinguished age: older adults reported feeling less bored and less fatigued. No differences by age were observed for any of the high arousal negative emotions (viz., anger, anxiety/worry). We expect that this may be due, in part, to a relatively lesser reports of negative emotions. Notably, these age differences in emotional experience held when taking into account physical health and cognition, as well as sleep patterns and individual difference in morningness.

Considering time of day, even more complexity was observed. Participants generally felt more negative and less positive in the evenings than they did in the mornings. However, interactions between age and time of day did not follow a conceptually meaningful pattern. The age benefit for relaxation was stronger in the mornings than evenings, for example, but this interaction was not observed for calm or content. In addition, older adults were more likely to report increased enthusiasm compared to younger adults in the evening, but other high arousal positive emotions did not show this same trajectory. The time of day effects also varied somewhat based on demographic factors. Only women showed a decline in emotional well-being across the day (i.e., less positive emotion and more negative emotion in the evening than in the morning). In addition, the increase in negative emotional experience at the end of the day was attenuated in old age for African Americans and blue-collar workers, but not for European Americans and white collar workers. Thus, the present set of findings leave answers to many questions about time of day equivocal. Still, they suggest that some of the seeming inconsistencies across findings related to aging and emotion might be at least partially be explained by the specific emotions sampled and time of day. Measures that focus exclusively on high arousal terms (e.g., PANAS) can be expected to find weaker age differences, missing important age-related increases in lower arousal positive emotion, perhaps especially so if sessions are run later in the day.

Past work based on experience sampling suggested that age differences are weaker for emotional intensity than emotion frequency (e.g., Carstensen et al., 2000, 2011). Carstensen et al. concluded that although older adults may experience negative emotions less often, when they do experience these emotions they are felt just as intensely. In the present study, however, we observed differences in intensity of emotional experience in the mornings and evenings and the pattern of findings did not change when we analyzed frequency instead of intensity. Unlike Carstensen et al.'s experience sampling project, in the present study, we compared only two time points: emotional experience upon waking in the morning and immediately before bedtime. One of the advantages of focusing on waking and bedtime is that age differences in environmental factors are likely smaller than in the middle of day (when younger and older adults engage in very different types of activities). Still, many interesting experiences in life occur in between mornings and evenings (indeed, the most intense emotional experiences likely occur then), and age differences may be more pronounced mid-day (when older adults have relatively more opportunities to be selective).

Differences in findings as a function of sample size and related statistical power also merit thoughtful consideration. Studies that rely on very large samples (e.g., Mroczek and Kolarz, 1998; Stone et al., 2010) are more likely to find significant age differences because they have the statistical power to detect very small effects. For instance, the widely reported increase in positive emotion observed by Mroczek and Kolarz (1998) was not meaningfully different from the non-significant increase reported by Carstensen et al. (2000, 2011). Thus, some apparent difference in findings about age-related shifts in emotion reflect statistical power. Similarly, in the present study and prior experience sampling research from our group, we observe very low rates of high arousal negative emotions regardless of age. Non-significant findings in frequency and intensity may mask other potential differences in the nature and influence of such emotions in daily life.

In the present study, age was largely unrelated to the experience of high arousal states, regardless of valence. For instance, age differences in negative emotional experience were driven by less intense, low arousal emotions, rather than declines in anger or anxiety. Reasoning from the vantage point of SAVI (Charles, 2010), low arousal emotions may be easier to manage and, thus, older adults are more successful in regulating them. This interpretation is also consistent with findings that suggest a lack of age differences in high arousal positive emotions (Lawton et al., 1992; Kessler and Staudinger, 2009; Scheibe et al., 2013). Scheibe et al. (2013), for example, found that older adults reported a greater preference for experiencing low arousal positive emotion relative to high arousal positive emotion than did younger adults. Whether or not high and low arousal emotions show distinct developmental trajectories and, if confirmed, the reasons for such differences remains highly speculative and demands targeted investigation.

Overall, the findings from the present study underscore the importance of carefully considering the emotions sampled and the time of assessment when examining emotional experience. Age-related changes in emotional well-being may be more pronounced for certain emotional states and at certain times of day.

### Conflict of interest statement

The authors declare that the research was conducted in the absence of any commercial or financial relationships that could be construed as a potential conflict of interest.

## Footnotes

^1^Yesterday interviews asked respondents “Did you experience [emotions] a lot yesterday?”

^2^In our prior experience sampling work we report frequencies based on endorsements greater than 1 for specific emotions (Carstensen et al., 2000, 2011). A relatively similar pattern of findings emerged when using this frequency index rather than the intensity based one we focus on here. The most notable difference was fewer interactions between Age and Time of Day (the only one that remained significant was for enthusiasm). In addition, there were no longer Time of Day effects for pride, sadness, and anxiety, but all the other Time of Day effects remained significant and a new one emerged, namely contentment was reported more often in the morning than in the evening (γ = −0.0125, *p* < 0.05). Importantly, all the age effects remained significant except for happiness (*p* = 0.16).
